# A Multi-Robot Sense-Act Approach to Lead to a Proper Acting in Environmental Incidents

**DOI:** 10.3390/s16081269

**Published:** 2016-08-10

**Authors:** Jesús Conesa-Muñoz, João Valente, Jaime del Cerro, Antonio Barrientos, Angela Ribeiro

**Affiliations:** 1 Centre for Automation and Robotics, CSIC-UPM, Arganda del Rey, Madrid 28500, Spain; jesus.conesa@csic.es; 2 Centre for Automation and Robotics, UPM-CSIC, José Gutierrez Abascal 2, Madrid 28006, Spain; joao.valente@upm.es (J.V.); j.cerro@upm.es (J.d.C.); antonio.barrientos@upm.es (A.B.); 3 Department of Systems Engineering & Automation, Carlos III University, Av. Universidad 30, Leganés 28911, Spain

**Keywords:** multi-robot sense-act system, collaborative robots, mixed robot fleet, site-specific treatment, precision agriculture

## Abstract

Many environmental incidents affect large areas, often in rough terrain constrained by natural obstacles, which makes intervention difficult. New technologies, such as unmanned aerial vehicles, may help address this issue due to their suitability to reach and easily cover large areas. Thus, unmanned aerial vehicles may be used to inspect the terrain and make a first assessment of the affected areas; however, nowadays they do not have the capability to act. On the other hand, ground vehicles rely on enough power to perform the intervention but exhibit more mobility constraints. This paper proposes a multi-robot sense-act system, composed of aerial and ground vehicles. This combination allows performing autonomous tasks in large outdoor areas by integrating both types of platforms in a fully automated manner. Aerial units are used to easily obtain relevant data from the environment and ground units use this information to carry out interventions more efficiently. This paper describes the platforms and sensors required by this multi-robot sense-act system as well as proposes a software system to automatically handle the workflow for any generic environmental task. The proposed system has proved to be suitable to reduce the amount of herbicide applied in agricultural treatments. Although herbicides are very polluting, they are massively deployed on complete agricultural fields to remove weeds. Nevertheless, the amount of herbicide required for treatment is radically reduced when it is accurately applied on patches by the proposed multi-robot system. Thus, the aerial units were employed to scout the crop and build an accurate weed distribution map which was subsequently used to plan the task of the ground units. The whole workflow was executed in a fully autonomous way, without human intervention except when required by Spanish law due to safety reasons.

## 1. Introduction

Search and monitoring are often preliminary stages when environmental incidents happen and usually help success in solving or alleviating the problem quickly. Nevertheless, continuous explorations covering large areas are really challenging in rough terrains constrained by natural obstacles. In these contexts aerial vehicles are proper devices due to their ability to access and easily cover large surfaces whereas they suffer from lack of acting capabilities. Therefore, acting tasks in an environmental incident can be split into two stages: (1) aerial inspection that can be accomplished with aerial vehicles and (2) ground intervention with more powerful platforms, which could be robotic. Thus, aerial inspection may provide a quick assessment of the affected areas to plan the ground intervention so as to be more efficient. This separation leads to multi-robot solutions made up of heterogeneous platforms, e.g., aerial and ground or sea units, that help to perform more efficiently in many environmental disaster scenarios, such as oil spills [1,2], forest fires [3,4] or earthquakes [5], or simply for large area surveillance [6]. Nevertheless, these combined solutions require some way to integrate both stages, inspection and actuation, in a fully automated way so as to efficiently perform the overall task. Some works already benefit from the advantages of multi-robot solutions, although most of them are partially dependent on human intervention. For example, in [7] a mixed fleet is proposed where aerial mobile robot is used to provide a global coverage during an area inspection, whereas the ground mobile robot is used to provide local coverage of ground features. Nevertheless, human intervention is required to provide waypoints to the ground fleet. Similarly, considering forestry surveillance, there are systems that currently use aerial units for fire detection although they still have not integrated the ground intervention in the overall system [8]. All these systems contain several manual steps and this high human dependency reduces their productivity so far. For example, in [9] some steps require three human operators. Thus, it would be very helpful to rely on an overall system that is able to automatically sequence the different stages without any human intervention, i.e., a system in charge of automating entirely the overall task workflow.

In this work, a centralized approach to automatically manage the multi-robot system workflow from a base station is proposed in contrast with the decentralized approaches explained in [10,11,12], more suitable when a precise coordination among units is required. The idea behind the present centralized approach is to have autonomous units with their own internal processing systems to deal with their single activities, but to coordinate them as cooperating fleets from an external base station, easy to transport, installed relatively close to the working area. In this manner, the computational load can decrease on the units, transferring the coordination task to the external computer allocated in the base station that has access to all the units.

Furthermore, most of the previous approaches are designed to work with specific platforms and tasks. Due to this, the idea of this work is to design a multi-robot system able to deal with any task and any platform, able to provide ordinary operations (initialization, pause, resume, stop, etc.). To do this, the proposed system will use generic controllers and workflows to decouple the generic actions (connection, plan reception, displacements, etc.) to perform the work and the specific operations to complete the task (take pictures, extinguish a fire, etc.).

One of the scenarios that would improve productivity by using the proposed approach is agriculture, in particular precision agriculture [13]. Precision agriculture proclaims the improvement of crop performance and environmental quality by crop management that considers spatial and temporal variability [14]. Site-specific weed management raises as one of the most important tasks in precision agriculture, which is based on the fact that weed populations are commonly irregularly distributed within crop fields and typically in patches so that chemical and/or physical weed control measures may be applied only where and when they are really needed. Thus, site-specific weed management has clear environmental benefits, mainly in extensive crops where research work reported that more than a 60% of herbicide could be potentially saved with the proper technology [15,16]. This represents a significant reduction of the impact that the agricultural practices have on the environment. According to [17], each Euro invested in pesticides, including herbicides, returns 4 Euros in crops saved. Considering that the total sales of pesticides in Europe is currently approximately 3390 million Euros per year [18], we can estimate that pesticides may provide over 13,500 million Euros per year in saved crops. The estimation does not consider the indirect, but substantial, environmental cost. In fact, it has been estimated that only 5% of pesticides reach the target weeds [19], whereas the bulk of each application (over 95%) is left to impact the surrounding environment. The economic value of pesticide environmental impact has been estimated to total approximately 8000 million Euros per year in the USA [17]. Approximately 50% of pesticide usage consists of herbicide treatments, to give an idea EU countries used approximately 135,000 tons of herbicides in 2007 [20].

A solution combining aerial and ground autonomous robots may mitigate the abusive use of herbicides. Indeed aerial inspection is suitable to obtain information about weed distribution and the crop state; essential data to optimally plan the intervention of the ground units in order to minimize the impact of the treatment task on both environment and crop [21]. Furthermore, multi-robot systems composed of several small/medium size robots show several advantage against conventional large vehicles as Table 1 summarizes.

The following sections describe the basic elements of the proposed multi-robot sense-act system, as well as the system developed to properly integrate the aerial inspections and ground actuation. Both inspections and interventions will be considered hereinafter as robot missions, therefore the software to properly integrate all the components of the multi-robot sense-act system will be denoted as Mission Manager. Finally, the results section summarizes the performance of the implemented multi-robot sense-act system during the tests carried out on a site-specific weed treatment.

## 2. Materials and Methods

### 2.1. Multi-Robot Sense-Act System

The proposed multi-robot sense-act system encompasses aerial and ground platforms that have to feature some minimal operation capabilities to allow task automation. These tasks are sent by high-level control systems by the Mission Manager as explained in next section. The basic capabilities required are summarized in Table 2. They correspond to those provided by most commercial outdoor mobile platforms [22,23,24,25]. Any complex inspection or intervention mission can be performed by properly sequencing these operations.

Apart from supporting operation execution, the platforms have to be endowed with sensory devices to acquire information about themselves and the environment. This information is very relevant because it is used by the Mission Manager to supervise the work execution and to keep everything under control. Considering outdoor operation, one of the most common sensors is a Global Positioning System or GPS, which will be in charge of providing with the unit position. This information is critical for safety reasons since it allows detecting whether the robots are following the scheduled trajectories or not.

Although aerial units usually rely on Inertial Measurement Units (IMUs), ground units are typically endowed with Attitude and Heading Reference Systems (AHRS) to determine its attitude (roll, pitch, and yaw angles), since less accuracy is required to know the inclination of the robot. Moreover, the heading of the ground robot, which is crucial when dealing inter-row tasks, can be also precisely estimated by using a two-antenna GPS system.

Aerial units must have additional sensors to perform the inspection. In fact, currently most commercial drones are equipped with visible or infrared cameras and GPS, allowing acquiring geo-referenced images to be latterly processed to extract relevant information.

Ground units have to rely on actuators to perform the intervention tasks assigned. Depending on them, onboard devices may vary considerably. For example, in case of aiming at carrying out an agricultural task, sprayers, seeders or harvesting robotic arms, among others, can be considered [26].

Finally, all the information provided by the units must be transmitted to a Control Centre or Base Station for monitoring purposes, which can be placed in a cabin next to the working area. The control station is usually equipped with powerful antennas and a router to create a wireless network to access the units and a computer that gathers and processes all the data.

### 2.2. Mission Manager

The Mission Manager is executed by the Base Station computer. It is the software application in charge of integrating and automating the aerial inspection and the ground intervention missions. Its main goal is to automate the working sequence required by the proposed multi-robot sense-act system. In general, although the robotic platforms used are fully autonomous and support the minimal operations summarized in Table 2, high-level software is required to coordinate the fleet. This coordination not only evolves the generation of the trajectories of the units to accomplish the missions and sending them to the platforms but also the fleet supervision while working. Thus, if an unexpected behavior is detected, it is automatically reported to the operator.

**Figure 1:**
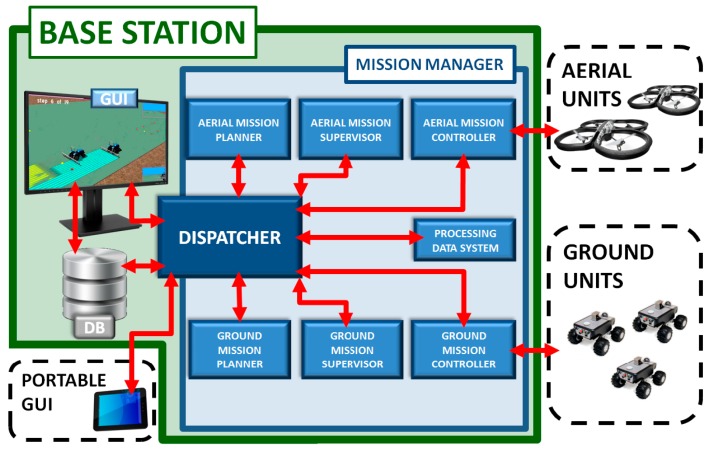
Architecture of the Mission Manager and its connections with external elements/systems.

Figure 1 shows the generic architecture of the Mission Manager.

In detail, the Mission Manager is composed of different modules:

- Aerial and a ground mission planners. They generate plans for units accomplishing the missions. Several planners are required for properly addressing the inherent differences of the aerial and ground units, as well as the different characteristics of inspection and intervention missions. For example, the ground units are usually limited by their turning radius, which constrain their trajectories, whereas the aerial trajectories may be limited by the battery capacity. The planners also have to take into account the features of the sensors onboard if they affect the trajectories. For example, if the aerial mission consists in taking pictures, the resolution and the size of the images of the camera must be considered in order to produce trajectories that allow capturing adequate pictures. Finally, the planners also have to consider aspects such as the fuel consumption, obstacles, distances, and unit speeds to guarantee safe and optimal trajectories.
- Aerial and ground mission controllers. They automate the mission at the fleet level. They provide an interface to communicate to all the vehicles as a coordinated fleet. They allow the vehicles in the fleet to execute simultaneously and cooperatively operations such as, launch, pause, resume, and stop. Moreover, controllers are dedicated to the decoding and transmission of the calculated plans that have to reach the units as an ordered set of commands supported by them. Additionally, the controllers must provide a stable channel to continuously receive the sensors information from the units. This information is critical to always keep the mission under control.
- Aerial and ground mission supervisors. They are in charge of monitoring and verifying that a mission is executed according to the plan. The mobile units’ environment is usually subject to unpredictable conditions such as wind, light changes, terrain roughness, and animals that may provoke small deviations from the scheduled plan. They receive the relevant unit’s information to analyze from the controllers. Once deviations are detected, supervisor systems should, at least, send a notification (alarm or warning) to the operator in charge of the fleet. The deviations are detected by comparing the current state of the units (position, speed, etc.) provided by the on-board sensors (e.g., GPS, IMU) and the previously calculated plans. If a difference large enough is detected, then an alarm will be generated to report the problem. The difference must be larger than some predefined thresholds to report real significant problems and to avoid noise and false positives. For example, the threshold margins employed on the results section to assume anomalies on trajectories and speeds were respectively 30 cm and 1 km/h. They help the human operator in charge of the mission to not miss any important detail.
- A processing data system, which receives and analyzes the data acquired in the inspection/scouting mission with the aim of extracting useful knowledge to the actuation/intervention mission. For instance, this module may consist of a mapping system to process images taken by the aerial units, detecting and obtaining the exact coordinates of the target points to be used for generating the plans for ground units.
- A dispatcher, which manages the entire workflow at the Mission Manager, integrating scouting, and intervention tasks. The dispatcher connects all the modules into the Mission Manager, redirecting processes to appropriate modules when it is required. Moreover, it manages external commands (plans, executions, pauses, resumes, and aborts) when the operator wants to directly control the workflow at the Mission Manager. The dispatcher allows the connection of new modules so that Mission Manager gets new functionalities.

In addition to the internal modules of the Mission Manager, the following external systems can be found in the proposed architecture:

- GUI (Graphical User Interface), which displays data generated by the Mission Manager such as plans, execution states, alarms, etc., guiding the operator through the different workflow steps. This module allows the operator to directly control to the Mission Manager modules and consequently gathers the human-system interaction. The main actions allowed are planning an aerial/ground mission, launching an aerial/ground mission, and processing the data acquired by the missions.
- Portable GUI, which allows operator to individually control the units outside the Base Station, i.e., at field. The portable GUI is actually useful in a breakdown case.
- Database, where all data such as plans, commands, or telemetry of the mission are stored. They are required to interrupt and resume the process, or even to perform offline processes when the units are not working, for example, the case of image processing or any other big data activity that could turn out to be important in the management of future incidents.

Due to their relevance, the mission controllers and the dispatcher are explained in detail in the following sections.

#### 2.2.1. The Mission Controllers

The proposed mission controllers automate the execution of missions through a high-level layer built based on the basic operations supported by the robotic units. In other words, they allow saving the gap between planning and supervision by allowing the automation of the cycle planning-execution-supervision. Specifically, the mission controllers are responsible for decomposing the mission plan into as many sub-plans as units are involved in the mission. The sub-plans contain a sequence of simpler and more understandable commands. For example, a sub-plan could be: (1) connect to the unit; (2) initialize it to execute the mission, for instance, setting the origin of the coordinates, the Base Station position, etc.; (3) send the sub-plan; (4) verify that it is successfully received; (5) wait until the mission is accomplished; (6) stop the unit; (7) confirm the unit is actually stopped; and (8) close the connection.

Since the automation sequences of mission controllers depend on events caused by many systems, such as requests from the GUIs, communication timeouts, confirmations from the units, connection errors, etc., as well as by previous events, such as if it has completed a similar request, if it is already connected, if a response was received, we can conclude that the mission controllers are reactive systems [27]. Therefore, their behaviors are difficult to be predicted because, at any moment, they respond to multiple asynchronous events based on their current state obtained from the previously received stimuli. The behavior of reactive systems is complex to analyze what makes them very prone to failure. Thus, it is essential to make a careful design, especially when they interact with potentially dangerous and expensive elements, as the robots needed in the case of an environmental incident.

**Figure 2:**
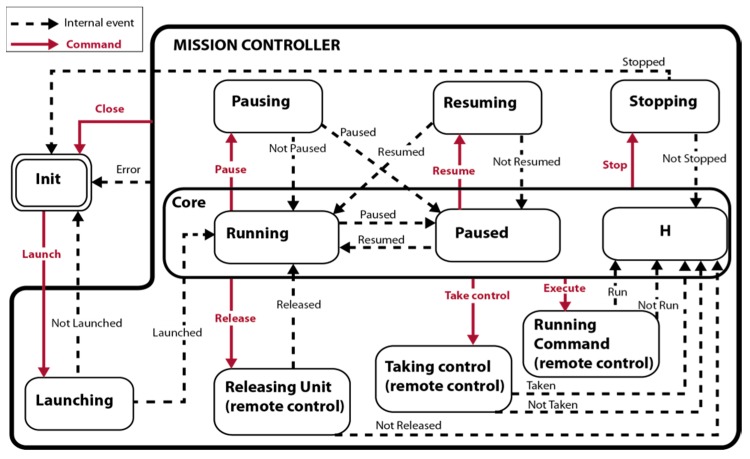
State diagram of a mission controller.

The main problem in the design of reactive systems lies in the difficulty of specifying the reactive behavior in a formal and rigorous manner. One solution is to use state diagrams in the design and development, such as those based on the *statechart* model [28], since the semantic richness of this model allows getting understandable visual representations. Therefore, assuming a set of basic operations common in drones and ground autonomous robots as shown in Table 2, the state diagram for a generic mission controller is proposed in Figure 2.

According to the *statechart* model, the “H state” represents the last state within a super-state. In case of Figure 2, “H state” may represent a *running* or *paused* state.

The high-level commands, in red in Figure 2, are the following:

- Launch a mission: The controller establishes with all the units and sends the corresponding sub-plan to each one using the actuation commands shown in Table 2. When a unit starts executing the assigned sub-plan, the controller emits the *launched* signal and changes its state to *running* state. If no unit starts, the controller emits the signal *not launched* and returns to the initial state.
- Pause a mission: The controller send a *pause* command to all units and change its state to *paused* state after checking that all vehicles have fulfilled the order. Otherwise, the controller outputs the signal *not paused* and remains in the *running* state.
- Resume a mission: The controller commands to continue the execution paused to every unit. The controller returns to the *running* state when at least one unit resumes the execution.
- Stop a mission: The controller sends a stop command to all units. When all units perform this command, the mission ends, the connections are closed, and the controller returns to the initial state.
- Close connections: This command closes all open connections when the units are already stopped.
- Take control of a unit (for remote operation): This command lets the operator to take the control of a unit of the fleet that is involved in the mission. The controller stores the remaining sub-plan before stopping the unit. The unit is no longer considered part of the fleet.
- Release a unit: It returns the unit to the fleet. The unit resumes the sub-plan stored by the controller when the unit was taken for remote operation.
- Run Command (for remote operation): It allows an operator to directly send commands (displacement, device setting, etc.) to a unit that is no longer part of the fleet and, therefore, is not involved in the mission.

Moreover, since many of the commands act on the entire fleet (launch, pause, resume, stop, and close), the controller must keep a track of each unit state, to determine when the internal signals or events that change the state of the mission occur (dashed lines in Figure 2). Note that, although many events are caused by external systems such as the requests received from the GUI or the paused/resumed signals received from the unit controllers in the lower level, there are also internal events caused by the mission controller that arise when a higher level situation is detected. For example, when all the units have sent the paused or the resumed confirmation, the mission controller assumes internally that the mission is paused or resumed, respectively, and emits an internal signal to switch to the corresponding state.

**Figure 3:**
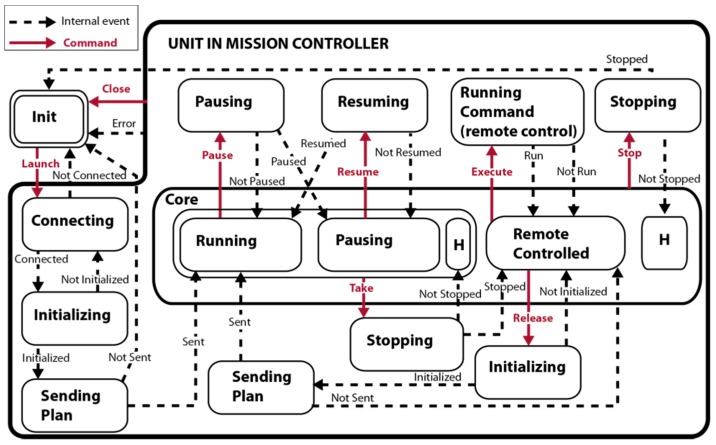
The state diagram of a unit controller for a mission.

Some of the proposed controller commands must be broken down into a sequence of several lower level commands. This is the case of operations, such as the launch of a mission and the release of a unit, resulting in two requests; first the initialization of the unit and second the sending of the mission sub-plan. It is therefore necessary to create a lower-level layer formed by three controllers, one per unit, called unit controllers for a mission. These controllers are responsible for interpreting the orders of the mission controller and recoding them based on the command repertoire of the unit. Figure 3 shows the state diagram of one of these controllers that is very similar to that presented in Figure 2. The main difference is that internal signals, in dashed lines, now are independent of the fleet and depend on a single unit. In addition, *launch* and *release* operations are separated into several steps to express in terms of low-level operations that can be executed by the units.

**Figure 4:**
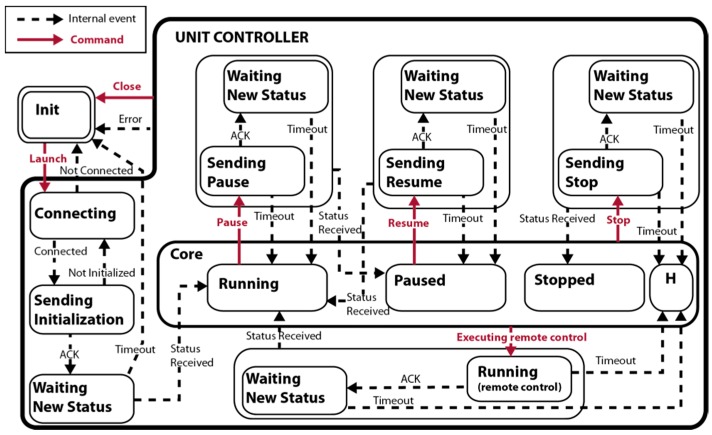
State diagram of the basic controller of a unit.

Since units, in general, are only able to confirm command reception without guaranteeing their execution, an additional layer is required to provide the system with a confirmation service of the order executions. Thus, this new controller (see Figure 4) implements the execution confirmation service based on two facts: (1) the units are able to report the command receipt by an acknowledge (ACK); and (2) the units periodically produce certain status information such as their internal state, e.g., speeds, positions, health, etc. This information comes from the sensors readings and it is analyzed to detect whether a relevant event has happened.

Basically, every time the controller receives a request, it is processed and the related lower level request is sent to the unit. Once the ACK message is received from the unit, the controller commutes to a new state where it waits for some new status that corroborates the execution of the requested operation by the unit. In the case of detecting a change, the new status is reported by issuing an internal signal and changing to the new corresponding state, otherwise, after elapsing a predefined time, the controller assumes that the request was not executed and a timeout is emitted.

Thus, it is possible to build a generic high-level controller for missions by using this multi-layer system that allows different complexity. It will be able to govern a fleet of several units with precision in the response, i.e., knowing whether the command was received or not in the units and whether it was executed successfully. In other words, a controller that provides execution confirmation service.

Note how the individual units can influence the global mission controller. They send their sensor readings to the individual unit controllers, which process the information and react by changing their internal states (connecting, running, paused, stopped, waiting for something, etc.) and issuing the corresponding signals (connected, running, paused, resumed, stopped, etc.) when they detect these events have happened. These signals/events are the inputs for higher controllers that similarly react to them. In the end, they influence the global mission controller and change their internal states.

Note that these diagrams can represent the sequences of actions and the transitions, but they cannot easily represent a timing constraint, such as to complete the task within some fixed time. With the proposed architecture, the supervisor module is in charge of taking care of these constraints. Since it runs simultaneously to the mission it can check whether the task is performing according to the expected schedule.

#### 2.2.2. The Dispatcher

**Figure 5:**
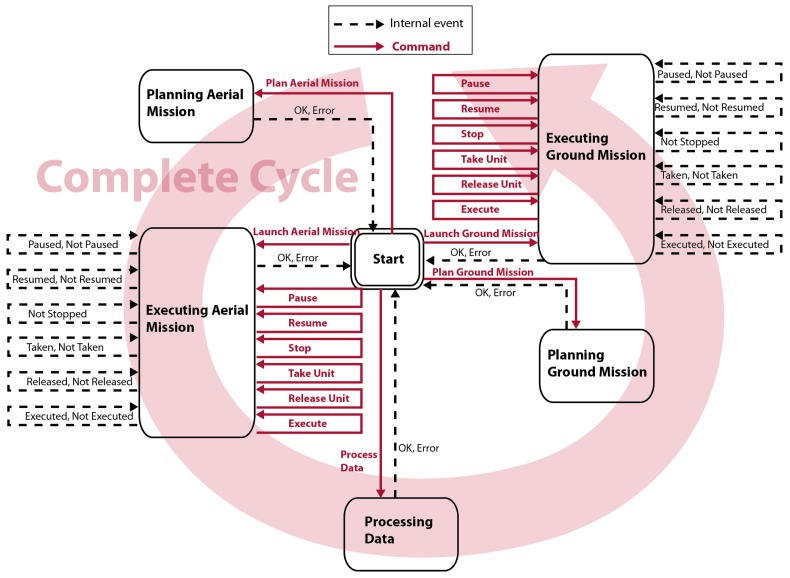
State diagram for the dispatcher.

The dispatcher is the distributor module of the Mission Manager. It contains the logic needed to redirect requests coming from the GUI or the portable device, creating the communication channel with the target module. It concentrates all communications with the internal systems of the Mission Manager by building a layer of abstraction that can replace any of the systems without having to make changes in the interfaces. The human-system interaction is centralized through this module. The interactions allowed are to plan an aerial/ground mission, to launch the mission, and to process the data acquired by the mission. The proposed dispatcher workflow can be expressed by the state diagram shown in Figure 5.

Within the states associated to the planners and the data processing, it is only needed to call the related systems (aerial/ground planners or the associated data process) and wait for their response. On the contrary, within the states associated to the missions, both the mission controllers and mission supervisors are employed. All information provided by controllers is redirected to the supervisor systems for being analyzed and detecting any anomalies. Note that if the full cycle is executed, the aerial and ground missions are properly combined.

## 3. The Multi-Robot Sense-Act System to Perform a Site-Specific Weed Treatment

The proposed multi-robot sense-act system was developed into the European project RHEA [29] to automate site-specific treatments. Within this project, two drones were used to inspect fields and three fully automated tractors to carry out the treatments. The fleet units and the internal Mission Manager modules employed are described below.

### 3.1. Aerial Fleet

**Figure 6:**
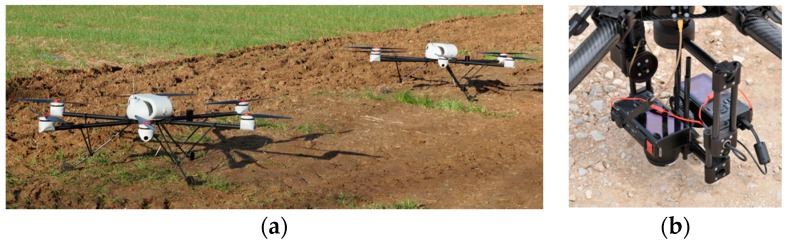
AR200 drones (**a**) with a detail of the camera mounting (**b**).

The aerial fleet was made up of two six-rotor drones (AR200 model), developed by the AirRobot company [30], each one with a flight autonomy of around 40 min. Six-rotor units were used to provide certain safety redundancy in case of failure in one motor. Drones were able to carry a sensor-payload up to 1.5 kg. They were equipped with two cameras, visible and near infrared spectrum; two Sigma DP2 Merril models, one of them modified to record NIR (near-infrared) images. The cameras were mounted on a gimbal system (see Figure 6) to reduce vibrations and to allow them to point down when the drones perform steady flights. Drones were able to provide telemetry information during the flight, including information required for supervision, such as position estimation and battery level.

The plans (mission) of the drones were an ordered list of points (way-points) where cameras had to take the pictures. The drones have to fly through the defined way-points, returning to their home points after finishing the mission.

### 3.2. Ground Fleet

**Figure 7:**
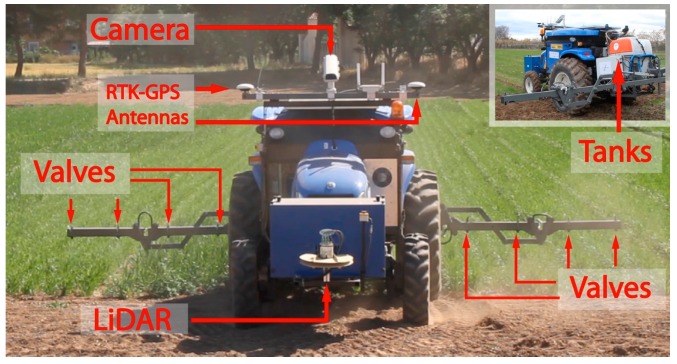
Ground unit.

The ground fleet was made up of three New Holland tractors of 50 hp and 1270 kg [31]. Each tractor (see Figure 7) was adapted by reducing the driving cabin and integrating onboard the vehicle the equipment required for the perception, actuation, location, communication and safety.

An RTK-GPS receiver system, an RGB camera and a LiDAR, were integrated to allow autonomous and safe navigation. The RTK-GPS receiver, a Trimble BX982 model, was a multi-channel, multi-frequency OEM GNSS receiver that provides with centimeter-level positioning to the navigation system. The receiver supports two antennas, which allows estimating the heading of the vehicle with high accuracy. Therefore, a single connection to the tractor receiver (via RS232, USB, Ethernet or CAN) provides both centimeter-accuracy positions and a heading that is accurate to less than a tenth of a degree (2 m baseline between the antennas). In this manner, both the position and heading of the vehicles are provided with high precision at a frequency up to 20 Hz.

The camera onboard each tractor was an SVS4050CFLGEA model from SVS-VISTEK (Seefeld, Germany) with a CCD Kodak KAI 04050M/C sensor and a GR Bayer color filter, which provides high-resolution images, i.e., 2336 by 1752 pixels with a 5.5 by 5.5 μm pixel size, to accurately locate in real time weeds, obstacles, and row crop lines The camera was placed inside a housing unit with a fan controlled by a thermostat for cooling purposes, which allows it to work even when raining or when the temperature is above 50 °C. The description of how onboard cameras detect weeds and the crop rows is out of the scope of this paper since this kind of detection is appropriate for wide-row crops, such as processing tomatoes, maize, strawberry, sunflower, and cotton [32]. Actually, the considered scenario only takes into account weed detection by remote sensing, since it is the best example to illustrate the integration of the whole elements of the fleet, i.e., the scouting mission with the intervention mission.

The LiDAR sensor was used to detect obstacles along the vehicle trajectory with a ground clearance of 70 cm. This sensor was an LMS 111 (SICK AG, Waldkirch, Germany) and was installed in the middle of the vehicle’s front with an inclination of 4°. This slight inclination was needed to detect short obstacles that may be in front of the units and 4° were selected because this value showed a good performance during the tests. Higher inclinations generated many false positives due to the plants and the soil.

To perform the treatment, the tractor was equipped with a selective sprayer bar of 6-m length with 12 nozzles, which could be independently activated, and two tanks, one to store water (around 200l) and another smaller one, to keep herbicide [33]. The sprayer was equipped with a direct injection system for mixing agrochemical and water just before opening a single or several nozzles, which reduces herbicide waste.

Additionally, the on-board computer, which executes the internal control system for managing the sensors and actuators as well as for allowing remote control, was a CompactRIO model 9082 from National Instruments. Similarly to the aerial units, this internal controller allows autonomous execution of some remote commands such as move, pause, resume, and stop, including performing a treatment plan. The plan is mainly composed of a list of the way-points the vehicles have to cover and also contains the states for the spraying bar, i.e., the nozzles that must be opened and closed, for each point and other mission parameters, such as speed. More details about the ground units and their capabilities, for instance navigation and implemented control techniques, can be found in [34].

### 3.3. Mission Manager Implementation

The aerial planner splits the field into smaller rectangles (the shape depends on the sensor of the camera) in an optimal manner considering the orientation of the field, the overlapping requirements for the pictures as well as the resolution required for image analysis. The result of this process determines the exact position (longitude, latitude, and height) where pictures have to be taken. Subsequently, it uses a Harmony Search algorithm to find the optimal order to cover them [35]. This algorithm is a meta-heuristic optimization method that finds the shortest trajectory (global optimum), that is, the order that produces the best harmony. The aerial controller and supervisor system are described in [36]. In this case the controller is divided into two modules: a high level module that decodes the plan generated by the planner and converts it into a sequence of commands supported by the drones and a low level module that sends the commands and interacts individually with each unit. The aerial supervisor analyzes all the information received by the low level controllers and checks that the mission is performing according to the plans, reporting to the operator unexpected situations by sending alarms to the GUI. Such alarms are generated, for example, due to a delay in the mission execution, a low battery level, or an inaccurate trajectory.

The ground planner splits the field into parallel tracks and deduces the best sequence to cover them. The planning for the vehicles of the fleet was formulated as an instance of the Vehicle Routing Problem (VRP) and addressed with a meta-heuristic method, in particular a Simulated Annealing approach [21]. The ground supervisor is explained in [37]. Basically it is a hierarchic structure composed of many simple supervisors, each of them, in charge of monitoring a low level behavior. In other words, there are several simple supervisors for testing the speed of each unit, their trajectories, the status of their actuators, etc. Regarding the controller, it was implemented from the concepts outlined in Section 2.2.1 and by using the state machine framework provided by the Qt libraries [38]. The three generic modules were adjusted to work with the vehicle of the RHEA fleet.

Since the approach was tested by spraying herbicide into the weeds it was needed to integrate a data processing module responsible for detecting weed patches from the images acquired by the drones. In this case, this module is divided into two subsystems: (1) a mosaicking system in charge of the composition and the orthorectification of the images [39]; and (2) a mapping system that detects and geo-references the weed patches [40]. This data processing system provides a weed distribution map, which is used by the ground planner to develop the plan of the ground fleet for effectively accomplishing the treatment.

The dispatcher is the module in charge of linking all the previous modules and was implemented following the state diagram proposed in Section 2.2.2 using the Qt state machine state framework. Finally, the GUI was developed using the robot simulation environment Webots [41]. The interaction between the system and the human operator plays an important role because, in such a system, there are a vast amount of events happening simultaneously and a human can easily miss critical information. To minimize this issue, the GUI was designed according to the strategy based on alarms proposed in [42]. The alerts are highlighted by using small pop-up windows or eye-catching tags that report the operator about the most recent relevant events, e.g., the start of a new unit, or even future events such as a collision warnings.

### 3.4. Base Station and Network

Aside from the aerial and ground fleet, the base station contains the main computer that executes the Mission Manager, which allows remotely managing the fleets and executing the processing services that evaluate the data acquired by the missions. It is a desktop computer with a motherboard ASUS Z87-K SK1150/PCX 3.0, Intel core i7 4771 3.5 GHZ CPU, 2 DDR3 1600 8GB PC3-12800 modules (16 GB RAM), and SSD with 240 GB. The system was powerful enough to allow simultaneous operation of all the active Mission Manager modules when performing a mission, the controllers, the supervisor system, as well as the GUI.

Additionally, the base station is equipped with antennas and a router to create a multi-technology wireless network. A network based on a single technology was discarded because ISM (Industrial, Scientific, and Medical) radio bands can be used without licensing and its performance may be impaired by devices not part of the communication system which may provoke congestion and interferences. That is, a non-privileged usage of the ISM radio band based on a single technology cannot guarantee the tight communication requirements in terms of latency, throughput, and connectivity that robotic control applications usually have. Consequently, a wireless QoS-enabled multi-technology network based on the simultaneous usage of multiple communication technologies (IEEE 802.11a, IEEE 802.11g, ZigBee PRO and GPRS) was adopted to improve the network robustness and performance as explained in [43]. This lowers the risk that the unpredictability of the wireless communication channel will disrupt the overlay communication as it is unlikely that transmissions over several technologies are affected at the same time. Also, deadline violations caused by congestion of the wireless channel can be reduced if the selection of communication technologies is coordinated among neighboring devices.

## 4. Results and Discussion

To test the multi-robot sense-act system developed, a winter cereal field was prepared containing weed patches. The idea was to autonomously and sequentially execute all the steps required to perform a site-specific herbicide treatment via the Mission Manager and with both aerial and ground fleets. A cabin was placed next to the field as Base Station. The cabin was equipped with a computer to run the Mission Manager and with a router and an antenna to create the wireless network and to connect to the units. The network latency was set to 4 Hz, i.e., the network provided the information acquired from the sensors every 250 ms. This frequency was enough since agricultural vehicles used in the test did not have to move at speeds higher than 6 km/h, which is a typical working speed for herbicide treatments.

**Figure 8:**
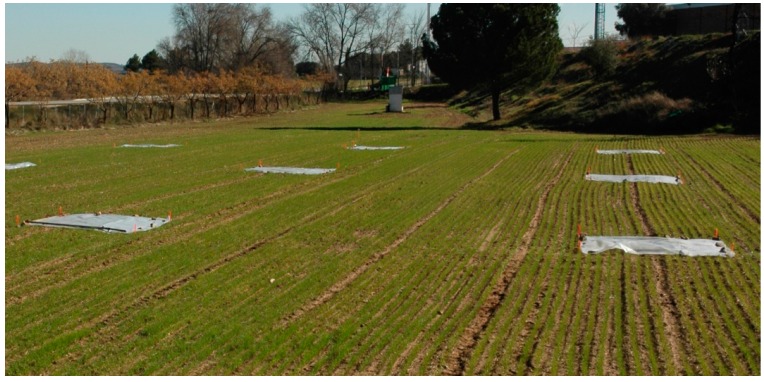
Winter cereal field prepared with nine seeded weed patches.

The platforms employed were the ones described in Section 3. The field was located in the experimental CSIC farm “La Poveda” (0°18′51.102″ N, 3°29′03.379″ W) in Arganda del Rey. It is 2400 m^2^ and was treated using a pre-emergency herbicide except for nine 3 × 3 m^2^ squares (see Figure 8), where some weeds (*Sinapis arvensis*) were seeded.

**Figure 9:**
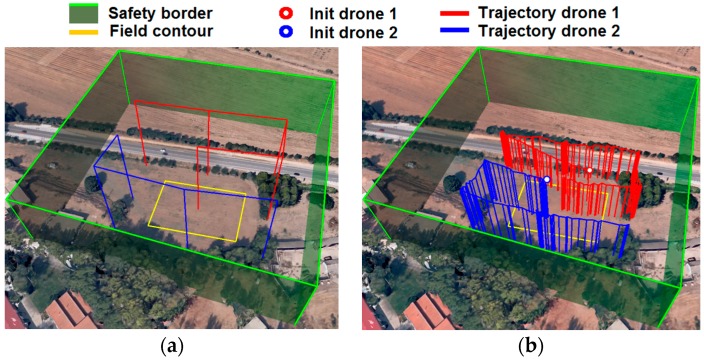
Inspection aerial mission: (**a**) Planned trajectories and (**b**) actual trajectories.

The field contour (yellow rectangle on Figure 9) was traced using a GPS and was stored in the database.

Initially, an aerial inspection plan was requested by using the GUI. Then, the aerial planner automatically built a safety border (green contour on Figure 9) expanding some margins of the field contour. This border could not be exceeded by the drones. Moreover, the aerial planner calculated the way-points where the drones had to take images for covering the whole field and taking into account the contour of the field, the flight attitude, the resolution, and the size of the images provided by the cameras. Images were used later to create the weed distribution map. Red and blue lines represented in Figure 9a stand for the route defined for each drone.

After defining the plan, the aerial mission controller requested the launch of the scouting mission, and the operator in charge of supervision approved the start of the mission. The plans were automatically loaded into the units and the supervisor pilot was asked (via GUI) to approve take-off. This step was carried out manually, even though the units had been able to perform it automatically in order to fulfill the guidelines from AESA, which is the Spanish agency in charge of establishing the aerial safety rules. The supervisor pilot raised the drones until the specified initial attitude. Then, the drones executed the inspection following the defined routes and yielding the trajectories shown in Figure 9b. The mission was monitored by the aerial supervisor system and non-failures were detected.

**Figure 10:**
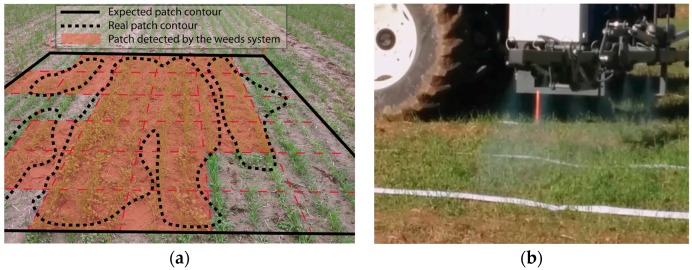
(**a**) Actual weed patch vs. expected weed patch and (**b**) paper strips along crop.

**Figure 11:**
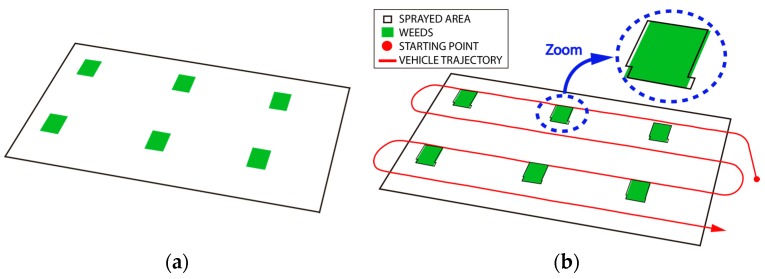
(**a**) Weed distribution map; (**b**) Ground mission plan and sprayed surface.

After concluding the plans, the Mission Manager requested a landing maneuver from the supervisor pilot so as to ensure that landing points were free of obstacles (people or machines). Once the cameras were on ground, their memory cards containing the images were manually removed and inserted into the computer running the Mission Manager. This was the only manual step accomplished during the whole process. The processing data system was invoked by the dispatcher. The system then provides a weed distribution map, which was used by the ground planner to develop the plan for treatment. Unfortunately, due to the weather conditions among others, the weeds did not grow as expected and did not have the shape of the expected patches (see Figure 10a). Consequently, the obtained distribution map, although it contained the real patch shapes, did not have the expected squares (compare shapes in Figure 10a), making it difficult to precisely determine whether the herbicide was sprayed on the appropriate areas. For this reason, the expected map was built manually to contain square patches (Figure 11a), even though the planner can address any shape (see patch shapes in mission performed in [44]), and was used to generate the treatment plan. The trajectories were optimized to reduce fuel use, so the ground planner generated a plan involving only a tractor.

The expected patches were covered with paper (Figure 10b). A total of five paper strips were used in each patch, arranged in parallel and spaced 1 m (three strips inside and two outside of the patch), for measuring the on/off time lag and therefore the percentage of the target area sprayed and not sprayed by water mixed with colorant. Then the treatment mission was executed very precisely; in fact, the real trajectories were nearly the same as the planned trajectories (Figure 11b). Only six of the nine patches were covered because the right part of the field was reserved for intermediate tests. The entire test can be watched on [45] as part of the RHEA project final demos.

**Figure 12:**
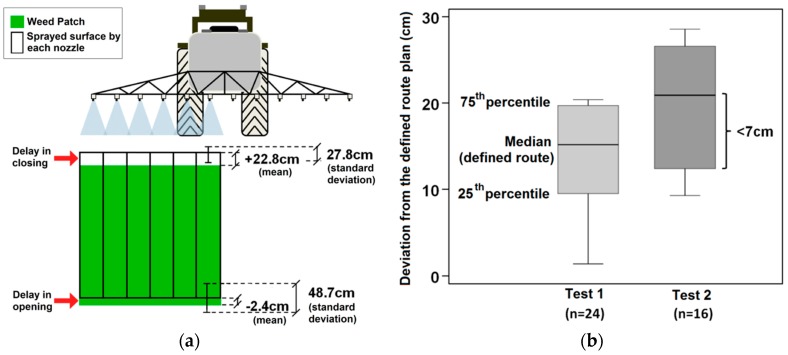
(**a**) Accuracy in the opening and closing of the herbicide spraying nozzles on the target area; (**b**) Differences between the distances from the actual trajectory of the UGVs and the planned path in the two tests performed.

An expert was asked to check the paper strips to see whether they had been sprayed or not and the results showed that the ground treatment mission successfully sprayed more than 97% of the target area, i.e., weed patches. Thus, there was no significant failure in the spraying operation. Nevertheless, slight delays in the opening and closing of the nozzles near the edges of weed patches were observed (Figure 12a) that were successfully detected and reported by the supervisor system. From an agricultural point of view, it is more important to correct the slight delay in the opening in order to adjust to a “conservative herbicide application”; that is, a slight advance in the opening of the nozzles can be tolerated in order to avoid leaving a part of the weed patch without spraying.

The assessment of the path followed by the tractor scarcely deviated from the defined route, indicating the high precision of the trajectory. Indeed, a deviation of less than 7 cm in the measures included between 25th and 75th quartiles regarding the median (i.e., the defined path plan) was observed in the two tests performed (Figure 12b).

It is important to note that, due to the sensors’ accuracy, the supervising systems included in the multi-robot approach are able to detect very small deviations in trajectories, speeds, or time of response of the valves and generate a huge number of alarms to the operator. However, the systems were configured to use thresholds so as to generate only alarms when some relevant deviations occur, avoiding in this manner to absorb the operator attention unnecessarily. Thus, the thresholds were set to 0.3 m for trajectory deviation, 1 s for the valve time response, and 1 km/h for speed.

Finally, a qualitative evaluation of the system by the operator in charge of the missions was done. This evaluation consisted in comparing the perceptions the operator had and what actually happened during the missions to see whether the operator perceived successfully the execution or not. The operator was asked to report whether or not he perceived an inappropriate working speed, an inaccurate trajectory, and any delay in the valves opening/closing. The operator said that he did not realize that the spraying had a delay before the system alerted him. Once the system reported this to him, he focused special attention on the spraying bar every time the tractor passed over the weeds and he realized that the delay, although small, was real. Regarding the trajectory the operator reported that the system generated many alarms during the turning maneuvres and he actually perceived that the tractor had some turning problems due to the mud in the headlands. Finally, the operator noticed visually some speed changes that were also alerted successfully by the system.

The same test was repeated 20 times to check the robustness of the system. Table 3 summarizes the average results and the main errors that happened during all these trials.

Only the missions that failed due to internal errors on the units were aborted. In such cases, the system detected that the units were not responding to the commands, reported to the operator, and aborted the mission. In the rest of the cases, the missions could continue even when some of the internal systems, such as the planners or the weeds system, failed. The system reported to the operator that, for example, the planner had failed due to some input parameter not properly specified and asked for a new path planning.

Regarding the missions that did not fail, the system also detected out of trajectory positions, valve delays, and wrong speeds in most of them. These errors appeared in the missions in the same way they appeared in the test explained above. They appeared in 80%, 75%, and 85% of the missions respectively. The wrong speeds and the out of trajectory positions were mainly due to the environment conditions (wind, mud, rough terrain...) and the valve delay to the low bar response time. As in the explained test, the operator corroborated that these failures actually happened during the missions.

Finally, 10 of the tests were performed with several tractors (see some demo in [46]) and in four of them, 40% of the missions, future collisions were detected by the supervising system and were neutralized by pausing/resuming some of the tractors involved.

A wider description of these tests and failures can be found in [37].

Note that, although the ‘out of trajectory’ and ‘wrong speed’ failures appeared in a lot of trials, there were occasional situations that were not present along the whole mission, just mainly on the headlands due to the mud. Additionally, every time they aroused the system, it reacted by alerting the operator, but note that the collision supervisor kept working to prevent any impact, that is, the supervising system is permanently watching to ensure the mission safety.

More tests were conducted in other fields, nevertheless they were not as complex and illustrative as the previous one. Their results can be found in other papers, however they only are focused on specific steps such as the aerial or the ground mission and do not cover the whole workflow. For example, the multi-robot system proposed was used to automate aerial missions and the results published in [36]. The system automatically linked the path planning, the execution, and the supervision. On the other hand, this system was also used for ground missions and some results were published in [37]. In this work, the steps related to the ground units were exhaustively tested. Thus, the path planning of several missions was carried out and the further trajectory executed by using the proposed system. Some aspects such as the vehicles’ speed, the trajectory accuracy, or the mission coordination were supervised to corroborate that they satisfied the mission constraints. More specifically, the overall system was able to perform the tasks without collisions in missions where several vehicles had to work cooperatively in the same workspace. Moreover, the vehicles performed at the expected speeds, mostly within a 1 km/h margin threshold, and accurately followed the trajectories within a 10 cm deviation margin. Additionally, the supervising module integrated into the proposed Mission Manager successfully detected those situations that the vehicles did not perform according to the mission requirements.

## 5. Conclusions and Future Work

A multi-robot sense-act system to properly combine autonomous aerial inspection and autonomous ground intervention missions to address environmental incidents has been proposed. The approach involves a heterogeneous, aerial and ground, fleet and a Mission Manager that allows a single operator to supervise the entire process and manage the workflow required to autonomously complete a mission composed of many different steps. The proposed system allows sequencing automatically all the steps by using platforms that support the usual minimal operations and for many general tasks in many environments, for example, in agriculture, where currently there is no system able to automatically link the data provided by the scouting missions into the treatments.

Indeed, due to this absence of automation, the proposed system has been tested by performing a real site-specific weed treatment, in which the scouting mission was used to acquire the data to detect the weed patch positions that allowed the intervention to treat only the infested areas and, consequently, reduce the cost of the treatment and the chemical pollution. The trial evaluated the integration between the aerial and ground tasks and yielded a spray accuracy of over 97% on the target area (weed patches) and a mean deviation lower than 7 cm from the planned route. Slight delays in the opening and closing of the nozzles at some border errors were observed and reported successfully by the ground supervisor module included into the Mission Manager. These delays can be corrected in the planning step by expanding the work area considered by the sprayer boom.

All the steps needed to achieve the site-specific weed treatment as well as the management of the workflow required to complete the entire process were entirely automated. Human intervention was only required to order the launch (take off) and stop (landing) of the aerial units (due to the current Spanish regulations) and to physically introduce the memory card containing aerial images into the Base Station computer, since the camera characteristics did not allow the real-time output of high quality images directly to the computer during the acquisition step. Additionally, the operator in charge of the mission reported that the system helped him to notice the spraying delays and confirmed that he visually detected some inaccurate speeds and maneuvers that were also detected and reported by the system.

In the future, cameras that allow image transmission to be automated could be tested although it is required that no image quality gets reduced in this process. This will make the human intervention necessary. The new approach will be tested for other types of scenarios.

## Figures and Tables

**Table 1:** Use of fleets of small-sized robots versus use of large platforms.

Aspects	Large Tractors	Fleets of Small/Medium Size Robots
Safety in autonomous operation mode	Becomes a safety problem in case of failure	Small/medium sized robots can interact with humans in a safer way
Fault tolerance	A failure will stop the entire work until the machine is repaired	Robot teams allow re-planning the overall task in case of failure of one unit
Impact on crop/field	High soil compaction	Lower soil damage (lighter vehicles) and more precise movement
Human Resources	One operator for each vehicle	One operator can supervise the whole fleet

**Table 2:** Minimal set of operations considered for both aerial and ground units.

Operation	Description
Initialization	Set up the initial configuration of a unit
Actuation	Actions on the unit (displacements, speed changes, tool activations, plan executions...)
Pause	Interrupt the current operation, keeping the same state until receiving a resume command
Resume	Resume the activity that was being carrying out when received the paused command
Stop	Stop the unit movement and actuation
Disconnect	Close the connection from which the request has been made

**Table 3:** Summary of trials results.

Failure	Importance (in Terms of Safety)	Missions Detected (%)	Mission Failed?	System Reaction
Units internal errors	Very high	15	Yes	Report the operator and abort the mission
Wrong path planning	Medium	0	No	Report and ask for a new planning
Mission not loaded	Low	15	No	Report and ask for a new execution
Weeds system failed	Low	0	No	Report and ask for a new execution
Out of trajectory	High	80	No	Report
Valves delay	Low	75	No	Report
Wrong speed	Medium	85	No	Report
Collisions	Very high	40	No	Report and manage the traffic (pause/resume the units that are going to collide)
